# The α-Synuclein Monomer May Have Different Misfolding Mechanisms in the Induction of α-Synuclein Fibrils with Different Polymorphs

**DOI:** 10.3390/biom13040682

**Published:** 2023-04-17

**Authors:** Nannan Zhao, Qianqian Zhang, Fansen Yu, Xiaojun Yao, Huanxiang Liu

**Affiliations:** 1 School of Pharmacy, Lanzhou University, Lanzhou 730000, China; 2 Faculty of Applied Sciences, Macao Polytechnic University, Macao SAR, China; 3 College of Chemistry and Chemical Engineering, Lanzhou University, Lanzhou 730000, China

**Keywords:** α-synuclein fibrils, template-induced misfolding, steered MD simulation, DCCM, secondary structure analysis, residue interaction network

## Abstract

The aggregation of alpha-synuclein (α-Syn) is closely related to the occurrence of some neurodegenerative diseases such as Parkinson’s disease. The misfolding of α-Syn monomer plays a key role in the formation of aggregates and extension of fibril. However, the misfolding mechanism of α-Syn remains elusive. Here, three different α-Syn fibrils (isolated from a diseased human brain, generated by in vitro cofactor-tau induction, and obtained by in vitro cofactor-free induction) were selected for the study. The misfolding mechanisms of α-Syn were uncovered by studying the dissociation of the boundary chains based on the conventional molecular dynamics (MD) and Steered MD simulations. The results showed that the dissociation paths of the boundary chains in the three systems were different. According to the reverse process of dissociation, we concluded that in the human brain system, the binding of the monomer and template starts from the C-terminal and gradually misfolds toward the N-terminal. In the cofactor-tau system, the monomer binding starts from residues 58–66 (contain β3), followed by the C-terminal coil (residues 67–79). Then, the N-terminal coil (residues 36–41) and residues 50–57 (contain β2) bind to the template, followed by residues 42–49 (contain β1). In the cofactor-free system, two misfolding paths were found. One is that the monomer binds to the N/C-terminal (β1/β6) and then binds to the remaining residues. The other one is that the monomer binds sequentially from the C- to N-terminal, similar to the human brain system. Furthermore, in the human brain and cofactor-tau systems, electrostatic interactions (especially from residues 58–66) are the main driving force during the misfolding process, whereas in the cofactor-free system, the contributions of electrostatic and van der Waals interactions are comparable. These results may provide a deeper understanding for the misfolding and aggregation mechanism of α-Syn.

## 1. Introduction

The progression of neurodegenerative diseases such as Alzheimer’s [1] is mainly due to the loss of neuronal structure and function. Misfolding and abnormal aggregation of some proteins significantly contribute to their cause, including Tau-protein, Prion-protein, and α-synuclein (α-Syn) [2]. α-Syn is an intrinsically disordered protein of 140 amino acids that is expressed in the central nervous system at presynaptic and perinuclear sites [3]. The α-Syn fibrils formed by the aggregation of the α-Syn monomer are responsible for certain neurodegenerative diseases called α-synucleinopathies, such as Parkinson’s disease (PD), multiple system atrophy (MSA), and Lewy body dementia (LBD) [4,5,6,7]. The α-Syn protein contains three regions: a lysine-rich N-terminus (residues 1–60), a hydrophobic region of non-β-amyloid components (also called the NAC region, residues 61–95), and the acidic C-terminal region (residues 96–140) [8,9,10]. With the development of crystallographic techniques, high-resolution α-Syn fibrils have been resolved by X-ray diffraction, cryo-electron microscopy, and nuclear magnetic resonance (NMR), revealing the diverse structural features of α-Syn fibrils [11,12,13]. The findings of Peng et al. [14] suggest that α-Syn fibrils may acquire various misfolded morphologies in different cellular environments and cause a variety of α-synucleinopathies. For example, the oligodendrocyte environment leads to the production of a glial cytoplasmic inclusion body (GCIs-α-Syn) strain in MSA, whereas pathological α-Syn aggregates in neurons as Lewy neurites in LBD (LB-α-Syn). The structure shows that GCI-α-Syn forms a more compact structure than LB-α-Syn. Meanwhile, Bousset et al. [15] have also demonstrated that two α-syn polymorphs show significant differences in their propensity to bind and penetrate cells, toxicity, and aggregation ability. Moreover, post-translational modifications and some cofactors, such as metals and macromolecules, also contribute to the structural diversity of α-Syn fibrils [16,17,18,19]. For example, Dasari et al. reported that tau protein can interact with certain regions of α-Syn to promote the formation of toxic aggregates [20]. The structures show that α-Syn fibrils formed in vivo and in vitro have different structural characteristics and the α-Syn filaments from the diseased human brain generally have stronger seeding ability than those formed in vitro [16,21].

How are the different morphological structures of α-Syn fibrils formed? How does the α-Syn monomer continue to misfold after the formation of the fibril nucleus? Moreover, how do cofactors affect the aggregation process of monomers? So far, the specific molecular mechanisms of these processes are not explicit. Therefore, it is of great value for understanding the pathogenesis of diseases to study the misfolding mechanism of α-Syn monomers under the template. However, it is difficult to observe the protein motion at the atomic level by traditional experimental methods, which can be supplemented by the molecular dynamics (MD) simulation method. Additionally, MD simulations have been widely applied for studying the unfolding and folding mechanisms of proteins and some unique properties of fibrils, such as tau, prion, and Aβ [22,23,24,25,26,27]. Moreover, it is also advantageous to show the detailed structural changes of the protein during the simulation [28,29,30]. In particular, the developed enhanced sampling MD simulation methods can greatly enhance the protein conformational sample, enabling observation of more rare events. For example, steered molecular dynamics (SMD) simulation can capture the dissociation process of the boundary chain in aggregates on the nanosecond time scale and have been widely used in studies of the dissociation process of proteins [31,32,33].

It is quite difficult to directly study the misfolding process of full-length α-Syn proteins. Therefore, in this work, we revealed the misfolding process of an α-Syn monomer induced by different templates by studying its reverse process, that is, the dissociation process of boundary chains from templates. Firstly, conventional MD simulations combined with Dynamic Cross-Correlation Map (DCCM) analysis were used to predict possible dissociation sites of α-Syn boundary chains from three different systems (α-Syn fibrils in diseased human brains, in vitro cofactor-tau induction, and in vitro cofactor-free induction). Then, SMD simulations were performed to explore the dissociation process of α-Syn boundary chains. The results show that three systems have different dissociation paths. In addition, the residue interaction network and energy analysis indicated that different α-Syn aggregation processes depended on different driving forces. These results can provide new insights into α-Syn fibril polymorphisms and explain the assembly mechanism of α-Syn fibrils in vivo and in vitro at the molecular level.

## 2. Materials and Methods

### 2.1. Conventional Molecular Dynamics Simulation

In this work, the 3D structures of three different α-Syn fibrils existing in diseased human brains, generated by in vitro cofactor-tau induction and created by in vitro cofactor-free induction, were obtained from the RCSB protein database (https://www.rcsb.org/, accessed on 7 May 2022)) (PDB ID: 6XYP [12], 7L7H [11], and 6SST [13], respectively). Polymers in the three models were cut to have the same residue length (residues 36–79) and capped at both ends for optimization with PyMOL version 1.5 [34].

All the simulations were performed by Amber 20 software package [35], and the protein was described using Amber ff14SB force field parameters [36]. Firstly, each system was placed in a cubic box filled with TIP3P water [37,38], and the distance between the edge of the box and solute was at least 12 Å. In order to maintain the electrical neutrality of the system, the chloride ions were added into the system [39]. In addition, the periodic boundary conditions were used to avoid edge effects [40]. Then, the steepest descent method combined with the conjugate gradient method were applied to minimize the energy of the system to eliminate the unreasonable atomic collision. Hereafter, the system was gradually heated from 0 K to 310 K under NVT ensemble. The coupling coefficient of a Langevin thermostat (companies/manufacturers, city (states abbreviation is required for USA and Canada), country) was set to 2.0 ps^−1^ to control the temperature [41]. Subsequently, under the NPT ensemble [42], six-step equilibration process was performed on each system to adjust the density of the solvent. Finally, 200 ns conventional MD simulation was performed under NPT ensemble for each system with the time step of 2 fs. During the simulation, the SHAKE algorithm [43,44] was used to limit the bond length involved the hydrogen atoms. The long-range electrostatic interaction was handled using the Particle Mesh Ewald (PME) [45,46] method with a cutoff of 10 Å.

### 2.2. Steered Molecular Dynamics Simulation

Steered molecular dynamics (SMD) is a computational method that applies external force to the system and drives the position change within a certain time, which can gain access to biologically relevant information related to non-covalent bonding [47]. In the simulation process, the speed of the force-applying atoms is constant, while the speed of the pulled atoms will be changed along with the influence of the resistance in the dissociation process. The force exerted on the pulling atom is determined by the velocity of pulling and the spring coefficient, which satisfies Hooke’s law:

(1) F(t)=2k[vt − x(t)],

where F(t) is the force on the pulled atom, k is the spring coefficient, v is the velocity of the force-applying atoms, and x(t) is the position of the pulled atom at time t. SMD is widely used for protein–small-molecule dissociation, protein–protein interactions, and it can provide an atomic-level description of the underlying event [48,49,50,51]. To investigate the specific dissociation mechanism for each model, a 10 ns SMD simulation was carried out using the last frame structure of the equilibrate simulation in conventional MD simulation as the initial structure. The vector direction of the mass center of the boundary chain (such as chain-1) and the mass center of remaining chains was set as the reaction coordinate. When the distance between the two centroids was greater than 20 Å, the boundary chain was considered to be completely dissociated. The final velocity (v) and spring constant (k) were first determined by a stepwise optimization method (Figure S1). Firstly, the k was set to 30, 40, 50, 60, and 70 kcal mol^−1^Å^−2^, respectively, while keep v fixed at 0.010 Å/ ps. The results show that the stiff spring was satisfied when spring constant was 70 kcal mol^−1^Å^−2^, 50 kcal mol^−1^Å^−2^, 60 kcal mol^−1^Å^−2^ for human brain, cofactor-tau, cofactor-free models, respectively. Here, all the SMD simulations were performed in Amber 20 software package [35].

### 2.3. Dynamic Cross-Correlation Map

Dynamic cross-correlation map (DCCM) Cij was introduced to judge the time-dependent motion of all Cα atom pairs [52], which has been extensively used to study protein dynamics [53,54,55]. The formula is as follows:

(2) $$C_{\mathrm{ij}}=\frac{1}{L}\sum_{1}^{L}\Delta R_{i}\left(t_{l}\right)\Delta R_{j}\left(t_{l}\right),$$

where L represents the number of frames; ΔR_i_ represents the change of spatial position of a certain residue or atom in two frames before and after; C_ij_ represents the correlation between the changes of a group of residues or atom vectors, with values ranging from −1 to 1. A value of 1 indicates that there is a strong positive correlation between the motion changes at two positions, which means that the direction of motion between the two residues is the same; −1 indicates that there is a negative correlation between the motion changes at the two positions; 0 means there is no correlation between the two changes.

### 2.4. Residue Interaction Network Analysis

Residue interaction network (RIN) is a two-dimensional network node diagram constructed by the three-dimensional structure of protein [56], which can identify different types of interactions between key residues. In this work, representative conformations at different stages were extracted during the dissociation process to construct RIN. The RING web server (https://ring.biocomputingup.it/, accessed on 20 June 2022) and the visualization software Cytoscape version 3.9.1 [57] were used to analyze the interactions between the residues.

## 3. Results and Discussions

### 3.1. Comparison of Truncated Structures and Non-Truncated Structures

**Figure 1:**
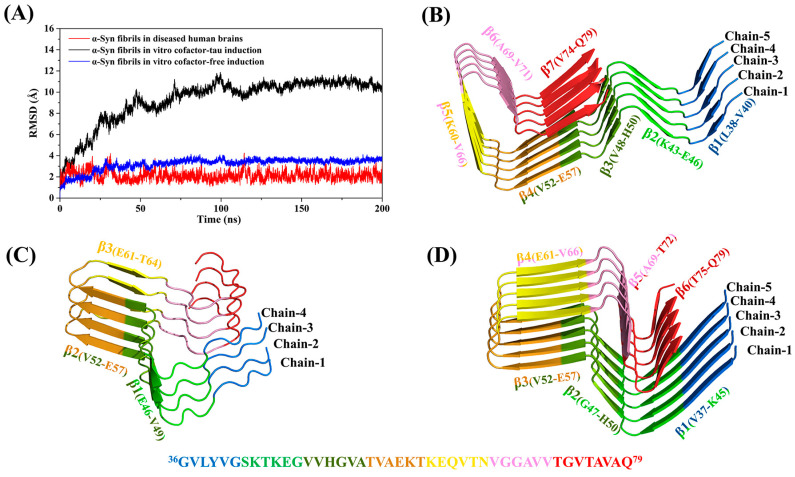
(**A**) The backbone RMSDs of α-Syn fibrils as a function of time in human brain (red), cofactor-tau (black), and cofactor-free (blue) systems. (**B**–**D**) show the 3D structures of the α-Syn fibrils in human brain (PDB ID: 6XYP), cofactor-tau (PDB ID: 7L7H), and cofactor-free (PDB ID: 6SST), respectively. The amino acid sequence is color-coded. The structures containing β-sheet are represented by β1–β7 in sequence.

To ensure that each α-Syn monomer in the three protein models contained the same number of residues, the structures from the diseased human brains and cofactor-free were truncated, but the most NAC core domain remained. Previous studies suggested that long-range interactions affect the stability of α-Syn [58,59], and the N-terminal or C-terminal truncation has a certain effect when the aggregation of α-Syn occurs [60,61]. Although our work is based on the structure of already-formed protein fibrils and may not have an impact, we still compared truncated and intact proteins to demonstrate that the truncation operation in this work does not affect the results. For convenience, we also numbered each chain in the polymers as shown in Figure 1.

Firstly, 200 ns conventional MD simulations were performed on both the truncated and non-truncated models, respectively. The root-mean-square deviations (RMSDs) of the protein backbone relative to the initial structure were calculated to monitor the convergence of systems (as shown in Figure S2). The results show that both the truncated and non-truncated systems reached the convergence states after 100 ns. It can be seen that the RMSD values of the truncated and non-truncated model of human brain were very consistent. However, the RMSD values in the cofactor-free model increased after truncation but the structure remained stable. Therefore, the DCCM and backbone hydrogen bond (H-bond) analysis was further performed based on the equilibrium trajectories to identify if the truncation affects peptide–peptide interactions. As shown in Figures S3 and S4, the truncated and non-truncated models from the human brain and cofactor-free both have similar results of DCCM and H-bond analysis, indicating that the truncation has no significant effect on peptide–peptide interactions. Subsequently, SMD simulations were also performed to further determine whether the truncation affects the dissociation pathway. As shown in Figure S5, the changes in the secondary structure of the boundary chain before and after truncation were also similar for the human brain model and the cofactor-free model. Therefore, it was appropriate to use the truncated protein structure for subsequent analysis in this work.

### 3.2. Preliminary Prediction of Initial Dissociation Sites for Boundary Chain by DCCM and H-Bond Analysis

Given that the dissociation and elongation of fibrils start from the boundary chain, the possible dissociation sites can be preliminarily predicted by studying the interaction between the boundary chain and adjacent chains. In the 200 ns conventional MD simulation, the RMSD values indicate that the simulation reached equilibrium at about 50 ns in the human brain and cofactor-free systems (Figure 1A). However, in the cofactor-tau system, the RMSD values were large (eventually stabilizing around 11 Å), and the system reached convergence after 100 ns. Therefore, the structure of the system derived from the human brain is the most stable. From Figure 1C, it can be seen that the structure of cofactor-tau contains more coil structures, so the RMSD values are large. In comparison, the structures from the human brain and the cofactor-free contain more β-sheets (Figure 1B,D), so their RMSD values are lower. In particular, although the RMSD values of the cofactor-free system are slightly larger than those of the human brain, its RMSD fluctuation is smaller due to its longer β-sheets. Overall, the RMSD results are consistent with the stability analysis of the initial structures of the three systems.

**Figure 2:**
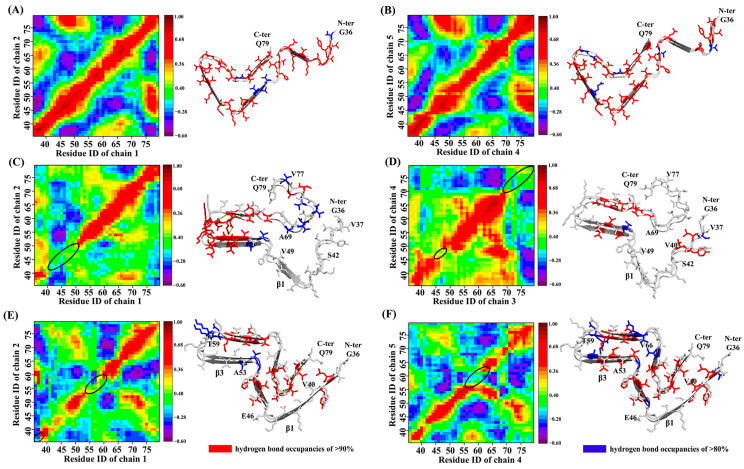
Analysis of DCCM (left) and backbone H-bond (right) in each system. (**A**,**B**) Chain-1/2 and Chain-4/5 in human brain system. (**C**,**D**) Chain-1/2 and Chain-3/4 in cofactor-tau system. (**E**,**F**) Chain-1/2 and Chain-4/5 in cofactor-free system. In DCCM analysis (left), the red region depicts a strong correlation between the residues, the blue region portrays a weak correlation and the black circles represent the possible initial dissociation sites. In H-bond analysis (right), red residues indicate the occupancy of hydrogen bond above 90%, blue residues above 80%, and white residues indicate weak H-bond interaction.

Then, the equilibrium trajectories based on each system were used for DCCM and H-bond analysis to reveal the peptide–peptide interactions between the boundary chain and its neighboring chain. First, through DCCM and H-bond analysis, it can be seen that in the human brain and cofactor-free systems, the boundary chains on both sides interacted similarly with their adjacent chains (Figure 2A,B,E,F), which indicates that the dissociation behavior on both sides may be consistent. In addition, the interaction strength between the boundary chain and the adjacent chain was strong at all positions in the human brain model, making it difficult to judge possible dissociation sites (Figure 2A,B). In the cofactor-free system, the interactions at the sheets of residues 40–46 and residues 53–59 were weak, so it may be the starting position of dissociation. At the same time, the C- and N- terminal may also be the initial dissociation sites (Figure 2E,F). In the cofactor-tau system, since both C- and N- terminals are coil structures, the environment of the boundary chains on both sides was not exactly the same. For example, in Chain-1/2, the C-terminal residues 69–77 had strong H-bond interactions, but they were not observed in Chain-3/4. Therefore, it can speculate that the starting site of the dissociation may be from the C- and N- terminals. In addition, residues 42-49 may also be the initial dissociation site (Figure 2C,D).

### 3.3. SMD Simulations Reveal Different Dissociation Paths in Three Systems

**Figure 3:**
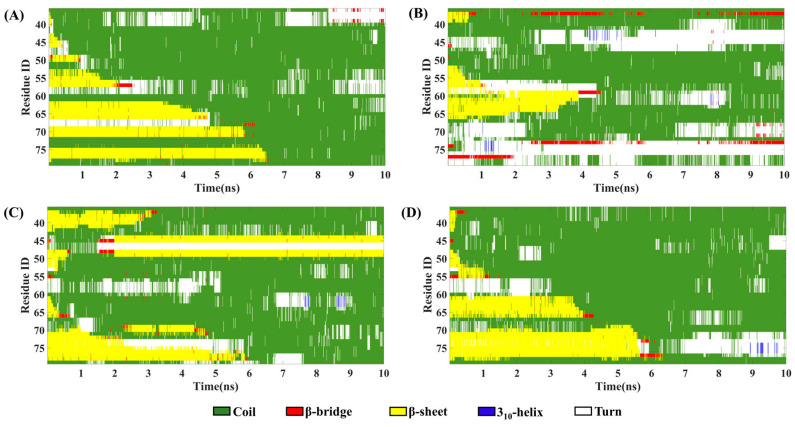
Changes in the secondary structure as a function of time. (**A**) Chain-1 in human brain system. (**B**) Chain-1 in cofactor-tau system. (**C**) Chain-1 in cofactor-free system for path1. (**D**) Chain-1 in cofactor-free system for path2.

**Figure 4:**
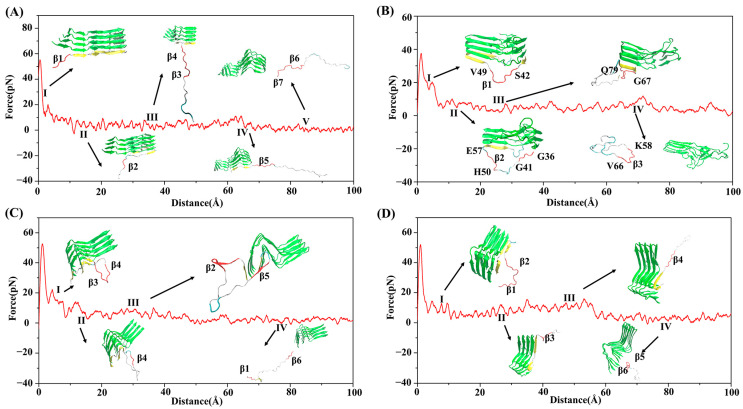
Changes of the pulling force over the reaction coordinate and the representative conformation of each stage. (**A**) human brain system; (**B**) cofactor-tau system; (**C**) cofactor-free system for path1; (**D**) cofactor-free system for path2.

First, to analyze the environment of the boundary chains on both sides, three parallel SMD simulations were performed on each system. The changes of force over the reaction coordinate (Figure S6) and secondary structure changes (Figure 3 and Figure S7) reveal that the dissociation processes on both sides were similar. Furthermore, the intermediate structures of the dissociation processes on both sides of the three systems were also similar (as shown in Figure 4 and Figure S8). Therefore, in the subsequent analysis process, we only focus on the Chain-1 side. Moreover, in order to ensure repeatability of the simulation, 10 SMD simulations were conducted for each system, respectively. At the same time, the secondary structure analysis on each trajectory was performed, and the results showed that there was only one path for the dissociation of the boundary chain in the human brain (Figure S9) and cofactor-tau systems (Figure S10). For the cofactor-free system, we found that there are two possible pathways (path1 and path2) for the dissociation of boundary chains. In order to make the results more statistically significant, 10 more dissociation trajectories (totally 20) for the cofactor-free system were further performed, 12 of which dissociated following the path1 and 8 dissociated following path2 (Table 1 and Figure S11). Finally, one of the trajectories was selected for each system for further analysis.

For the human brain system, the changes of secondary structure show that the β-sheet gradually transforms into a coil structure, and the transformation is complete at about 6.5 ns (Figure 3A). In addition, according to the change of the pulling force (Figure 4A), the whole dissociation process is roughly divided into five stages. The earliest dissociation region was residues 36–41 (contain β1, I), followed by the dissociation of residues 42–45 (contain β2, II). Subsequently, residues 46–57 (contain β3 and β4) dissociated in the third stage (III), followed by residues 58–66 (contain β5) in the fourth stage (IV). In addition, residues 67–79 (contain β6 and β7) dissociated in the fifth stage (V). It can be seen that the dissociation began at the N-terminal and proceeded sequentially to the C-terminal until the chain completely separated from the fibrils. For the cofactor-tau system, the complete conversion of β-sheet to the coil structure required a shorter time (about 4 ns) due to its lower β-sheet content (Figure 3B). The whole dissociation process can be divided into four stages (Figure 4B). The original dissociation sites were located at residues 42–49 (contain β1, I), which is consistent with the prediction in the CMD simulation. Next, the coil structures at N-terminal (residues 36–41) and residues 50–57 (contain β2) were dissociated (II). The third stage (III) was the dissociation of structures at the C-terminal (residues 67–79). Eventually, the remaining residues 58–66 (contain β3) dissociated from the fiber interface (IV). 

In the cofactor-free system, there were two different dissociation paths. As shown in Figure 3C, the residues 47–67 (contain β2/β3/β4) quickly transformed into coil in path1, followed by residues 36–46 (contain β1) and finally residues 68–79 (contain β5 and β6). At about 6 ns, the β-sheets were completely converted to the coil. For the dissociated β2, the β-sheet formed spontaneously at about 2 ns, but this had no effect on the subsequent dissociation process. The dissociation processes were divided into four stages (Figure 4C). First, β3 and the turn between it and β4 (residues 51–62) dissociated from fibril (I), followed by the dissociation of β4 (II). This was followed by the dissociation of β2 and β5 (III). Finally, β1 and β6 were dissociated in the fourth stage (IV). For path2, it was similar to the human brain system where the dissociation occurred sequentially from the N-terminal to the C-terminal, and the β-sheet also gradually transformed into the coil in 6 ns (Figure 3D). Like path1, the dissociation included four stages, that is, β1/β2 (I), β3 (II), β4(III), and β5/β6 (IV) (Figure 4D). The results show that the initial dissociation sites for both path1 and path2 were consistent with those predicted in the CMD.

### 3.4. Residue Network Analysis Reveal the Changes in Residue Interactions during Dissociation

**Figure 5:**
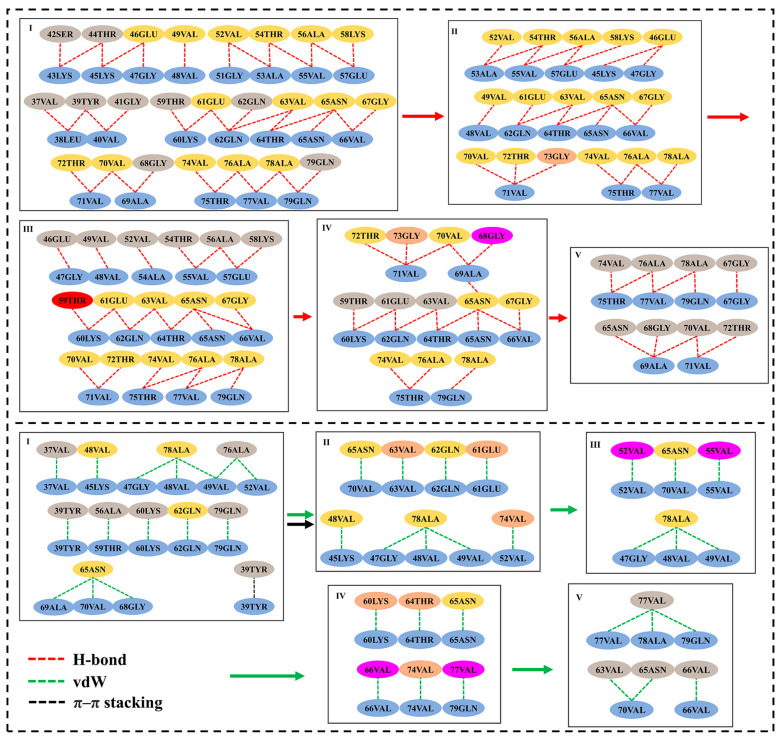
The residue interaction network analysis for human brain system. The yellow and blue ovals represent the residues of Chain-1 and Chain-2, respectively. The gray ovals indicate the residue to be dissociated at the present stage. Magenta ovals represent residues with increased interactions compared to the previous stage. The pink ovals represent residues with increased interaction compared to the previous stage but with dissociation compared to the next stage. The red ovals represent the residues that disappeared in the previous stage but reappeared in this stage. The red, green, black dash lines represent H-bond, vdW, and π–π interactions, respectively. The red, green, black arrows represent the processes of H-bond, vdW, and π–π interactions from one stage to another, respectively.

To further show the detailed changes in the residues’ interactions between boundary chains and fiber interfaces during dissociation, residue interaction network (RIN) analysis was performed based on the representative structure. Firstly, for the human brain system (Figure 5), the first stage was the dissociation of residues 36–41. At the same time, the H-bonds and van der Waals (vdW) interactions formed by these residues such as Val37 and Tyr39 (gray ovals in Figure 5-I) were destroyed. In addition, the π–π interaction between the Tyr39 residue pair was also destroyed, which indicates that Tyr39 was completely dissociated at this stage. Thereafter, the intermediate structure was stabilized by enhancing the H-bond interactions of Gly73 with Val71 and the vdW interactions of the Glu61, Val63, and Val74 residue pairs (pink ovals in Figure 5-II). However, these interactions were disrupted in the second dissociation stage (II), and the subsequent stabilization of the intermediate structure was achieved by increasing the vdW interactions between residues Val52 and Val55 (magenta ovals in Figure 5-III) as well as the H-bond interactions between residues Thr59 and Lys60 (red ovals in Figure 5-III). In the third stage, residues 46–57 were dissociated and the H-bond interactions formed by Glu46 and some other residues (gray ovals in Figure 5-III) were mainly damaged. The stability of the system was maintained through increasing the H-bonds’ interactions formed by residues Gly68 and Gly73, and vdW interactions formed by residues Lys60, Thr64, Val66, Val74, and Val77 (pink and magenta ovals in Figure 5-IV). The fourth stage (IV) was the dissociation of residues 58–66, which mainly destroyed the H-bond interactions formed by Thr59, Glu61, Val63 and Gly73, and the vdW interactions formed by Lys60, Thr64, and Val74 (gray and pink ovals in Figure 5-IV). The last stage was the dissociation of residues 67–79, in which a large number of H-bonds’ interactions and vdW interactions were both destroyed (gray ovals in Figure 5-V).

For the cofactor-tau system (Figure S12), the first dissociation stage was residues 42–49, the H-bonds’ interactions between some residues such as Gly41 and Thr44, and vdW interactions such as Val48 and Val49 were destroyed during dissociation (gray ovals in Figure S12-I). Subsequently, the intermediate was stabilized by enhancing the hydrogen bonding of Ala78 and the vdW interactions of Val74 and Thr75. Meanwhile, the stability of the fibril interface was mainly maintained by the new H-bond formed by Thr54, Glu61, and Thr64 (pink and magenta ovals in Figure S12-II). The second stage was the dissociation of residues 36–41 and 50–57. During this stage, the H-bonds’ interactions formed by Gly36, Val37, Tyr39, Ala53, Thr54, Val55, Gln62, Thr64, and Gly67 and the vdW interactions formed by Tyr39, Val40, and Asn65 as well as the π–π interaction between Tyr39 residue pairs were mainly damaged (gray and pink ovals in Figure S12-II). Meanwhile, the H-bond interactions between Lys58 and Chain-2 were increased to keep the stability of the whole protein. The third stage was the dissociation of residues 67–79, which was basically due to the destruction of the H-bonds formed by Glu57, Ala76, Ala78, and Gln79, and the vdW interactions formed by Val74 (gray ovals in Figure S12-III). The last stage was the dissociation of residues 58–66, which mainly involved the rupture of the H-bonds formed by Lys58, Lys60, Glu61, Gln62, Val63, and Asn65, and the vdW interactions formed by Glu61, Gln62, Thr64, and Asn65 (gray and pink ovals in Figure S12-IV). 

For the cofactor-free system, although there were two dissociation paths, the key interactions were similar (Figures S13 and S14). The π–π interactions formed by Tyr39 were destroyed in the fourth stage of path1 and the first stage of path II, respectively. The vdW interaction formed by Tyr39 was also disrupted in the fourth stage of path1, which indicates its importance. In addition, when the segments containing the dissociation of residues 58–66 dissociated from fibrils in the first two stages of path1 and the third stage of path2, respectively, the H-bonds and vdW interactions were damaged to an equivalent extent. In addition, the H-bonds’ and vdW interactions were also destroyed equally at every other stage of dissociation processes, which was consistent with the results of energy. Moreover, in the whole dissociation process, the formation and destruction of interactions that do not belong to the dissociation position rarely occurred, and therefore the whole fibril was relatively stable, which was different from α-Syn fibrils in the in vitro cofactor-tau induction but similar to the human brain system. However, its stability was lower than the human brain system but higher than the cofactor-tau system.

### 3.5. Misfolding Mechanism of α-Syn Monomer Induced by Different Templates

**Figure 6:**
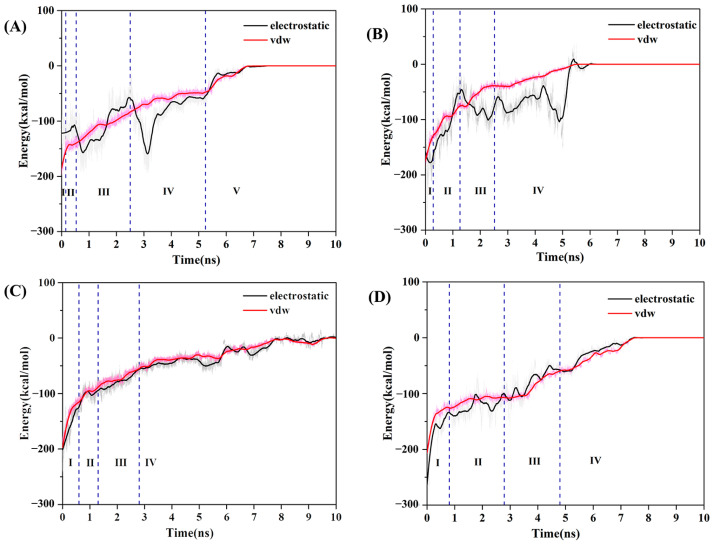
Changes of the energy between the boundary chain and template for (**A**) human brain system; (**B**) cofactor-tau system; (**C**) cofactor-free system in path 1; and (**D**) cofactor-free system in path 2. I–V represent the dissociation stages. The black lines represent electrostatic interactions, red lines represent van der Waals interactions, and blue dashed lines distinguish different stages.

**Figure 7:**
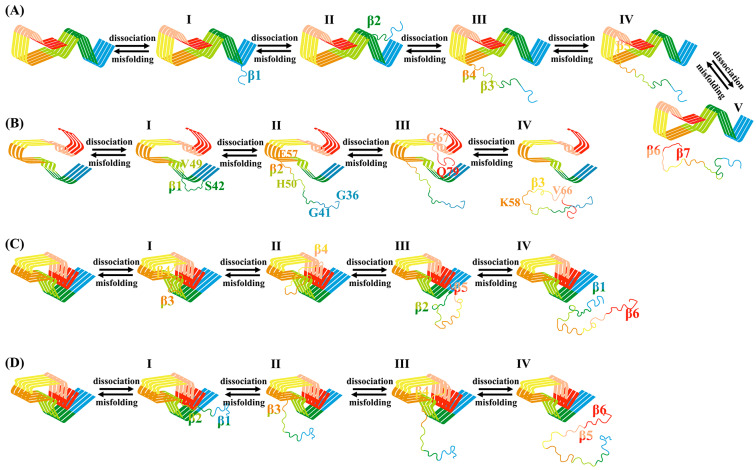
Schematic representation of the dissociation mechanism of the boundary chain and the deduced misfolding mechanism of α-Syn monomer in the induction of three different templates. (**A**) human brain system; (**B**) cofactor-tau system; (**C**) cofactor-free system in path1; (**D**) cofactor-free system in path2.

Since the dissociation of the boundary chain is the reverse process of template-induced monomer binding, the driving force for the continued misfolding of α-Syn monomers under template induction can be deduced by the resistance during the dissociation process. Therefore, changes in the contributions of the van der Waals (vdW) and electrostatic interactions between boundary chains and fibril interfaces during dissociation were analyzed (Figure 6). In the human brain system (Figure 6A), vdW interactions were mainly destroyed in the first two stages (I–II). In the third and fourth stages (III–IV), the electrostatic interactions were mainly destroyed. It is worth noting that there was a sharp peak in the electrostatic interaction energy in the fourth stage, which indicates that the dissociation of β5 (residues 58–66) needed to overcome stronger electrostatic interactions. Residues 58-66 contained more charged residues (such as Lys58, Glu61 and Lys60), so they tended to form H-bond interactions. In addition, it can also be seen from the above residue network analysis that the H-bond interactions were relatively dense in this stage (Figure 5-IV), which was also the reason why the electrostatic interactions became the main resistance. In the fifth stage (V), the electrostatic and vdW interactions were damaged in a similar degree. In general, the boundary chain was easy to dissociate from β1 at the N-terminal and then dissociate sequentially along the direction from β2 to β7 (Figure 7A). Conversely, the binding process of the incoming monomer with template was successively from C-terminal (β7) to N-terminal (β1). During the misfolding process, hydrophobic and electrostatic interactions first drove the binding of the α-Syn monomer to the template, then the electrostatic interactions stabilized the intermediates, and finally relied on the hydrophobic interactions to complete the misfolding process.

For the cofactor-tau system (Figure 6B), during the transformation from the second stage to the third stage, the dissociation of the boundary chain was mainly subjected to vdW resistance. In all remaining stages, however, it was predominantly destructive electrostatic interactions. Notably, the electrostatic interactions also had a sharp peak in the fourth stage (IV), and this stage was also the dissociation of residues 58–66 (contain β3), which indicated that residues 58–66 played an important role in the process of misfolding. Therefore, in the cofactor-tau system (Figure 7B), the binding process of the α-Syn monomer with fibril template proceeded from residues 58–66 and mainly relied on electrostatic interactions. Subsequently, the C-terminal structure (residues 67–79) also bonded to the template driven by electrostatic interaction. Next, the N-terminal coil structure (residues 36–41) and residues 50–57 (contain β2) were misfolded by electrostatic interactions and a small amount of vdW interactions. Finally, residues 42–49 (contain β1) completed the whole misfolding process driven by electrostatic interactions. In summary, both in the human brain and cofactor-tau systems, electrostatic interactions were the main resistance to boundary chain dissociation and vice versa the main driving force for monomer misfolding, especially residues 58–66. For the cofactor-free system (Figure 6C,D), whether path1 or path2, it can be seen that the energy contributions of vdW and electrostatic interactions were similar during the whole dissociation process. For path1 (Figure 7C), the misfolding started from the at the C- and N-terminal β1 and β6, followed by the adjacent residues (β2/β5), and finally the remaining residues (β3/β4). For path2 (Figure 7D), the misfolding process of the α-Syn monomer was similar to the human brain system, that is, it gradually misfolded from the C-terminal to the N-terminal. During the misfolding process, the electrostatic and vdW interactions were the driving forces for monomer-template binding, in both pathways.

## 4. Conclusions

In this work, the conventional MD simulation combined with steered MD simulation were performed to investigate the misfolding mechanism of α-Syn monomers in the induction of α-Syn fibrils with different polymorphs (diseased human brain, cofactor-tau and cofactor-free models). First, RMSD analysis showed that the α-Syn fibrils from the human brain were the most stable, whereas tau-induced structural changes were most evident. Next, the misfolding mechanism of the monomer was inversely deduced through the dissociation process of the boundary chain performed by SMD simulations. The results showed that the initial dissociation sites of the boundary chains in the SMD simulations were exactly consistent with those predicted by H-bond and DCCM analysis in CMD. For the human brain model, the dissociation process was successively from N-terminal to C-terminal. In contrast, the monomer’s binding occurred from the C-terminal (β7), followed by sequential folding toward the N-terminal (β1). For the cofactor-tau system, according to the dissociation path, it can be concluded that the binding of the monomer to the template started from residues 58–66 (contain β3), then residues 67–79, followed by residues 36–41 and residues 50–57 (contain β2), and finally residues 42–49 (contain β1). However, there were two different misfolding paths (path1 and path2) for the cofactor-free model. In path1, the monomer bonded first to the N/C-terminal (β1/β6), then to the adjacent residues (β2/β5) and finally to the remaining residues (β4/β3). In path2, monomers were combined sequentially from the C-terminal to the N-terminal, similar to the human brain system. In addition, energy analysis showed that in the human brain and cofactor-tau systems, the misfolding process of the monomer was mainly driven by electrostatic interaction; especially, the residues at 58–66 were the key positions in the folding process. In the cofactor-free system, no matter whether in the path1 or the path2 system, the energy contribution of electrostatic interaction and van der Waals interaction to the misfolding process was basically the same. In summary, this work reveals the misfolding mechanism of α-Syn monomers induced by three different α-Syn fibrils and elucidates the driving force of aggregation. New results provide a theoretical basis for understanding the pathogenesis of α-Syn.

## Figures and Tables

**Table 1:** Dissociation path statistics of the boundary chains of the three systems.

	Human Brain	Cofactor-Tau	Cofactor-Free
path1	10	10	12
path2	-	-	8
total	10	10	20

## Data Availability

Not applicable.

## Supplementary Materials

The following supporting information can be downloaded at: https://www.mdpi.com/article/10.3390/biom13040682/s1, Figure S1. Parameter correction for SMD; Figure S2. Convergence verification Titlie; Figure S3. DCCM analysis from converged trajectories of the CMD simulation; Figure S4. Backbone hydrogen bonds analysis from CMD simulation; Figure S5. Changes in the secondary structure as a function of time; Figure S6. The changes of force over the reaction coordinate; Figure S7. Changes in the secondary structure as a function of time; Figure S8. Changes of the force over the reaction coordinate; Figure S9. Changes in the secondary structure as a function of time; Figure S10. Changes in the secondary structure as a function of time; Figure S11. Changes in the secondary structure as a function of time; Figure S12. The residue interaction network analysis for cofactor-tau system; Figure S13. The residue interaction network analysis for cofactor-free system in path1; Figure S14. The residue interaction network analysis for cofactor-free system in path2.
