# Supplementary material for: The α-Synuclein Monomer May Have Different Misfolding Mechanisms in the Induction of α-Synuclein Fibrils with Different Polymorphs
Source: Biomolecules. 2023 Apr 17;13(4):682. doi: 10.3390/biom13040682 (PMC10136287; doi:10.3390/biom13040682)
Supplement: Supplementary file 1 [file biomolecules-13-00682-s001.zip › biomolecules-2237895-supplementary.pdf]

Supporting Information:

The α-Synuclein Monomer May Have Different Misfolding Mechanisms in the Induction of α-Synuclein Fibrils with Different Polymorphs

Nannan Zhao [1,†], Qianqian Zhang [2,†], Fansen Yu [1], Xiaojun Yao [3] and Huanxiang Liu [1,2,*]

[1] School of Pharmacy, Lanzhou University, Lanzhou 730000, China
[2] Faculty of Applied Sciences, Macao Polytechnic University, Macao SAR, China
[3] College of Chemistry and Chemical Engineering, Lanzhou University, Lanzhou 730000, China
* Correspondence: hxliu@mpu.edu.mo; Tel.: +853-8599-6874
† These authors contributed equally to this work.

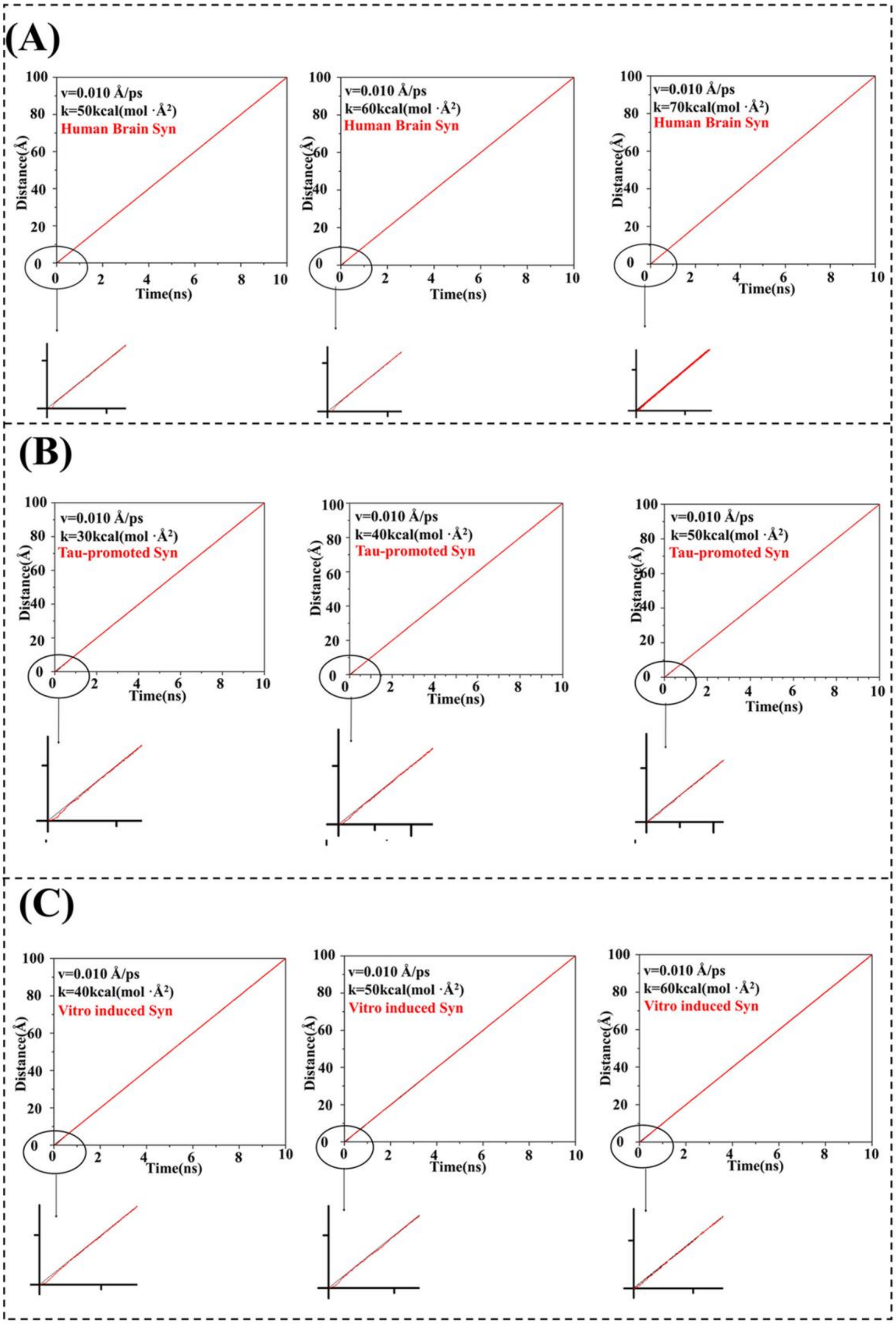

Figure S1. Parameter correction for SMD. (A) Diseased human brain model. (B) In vitro cofactor-tau induction model. (C) In vitro cofactor-free induction model.

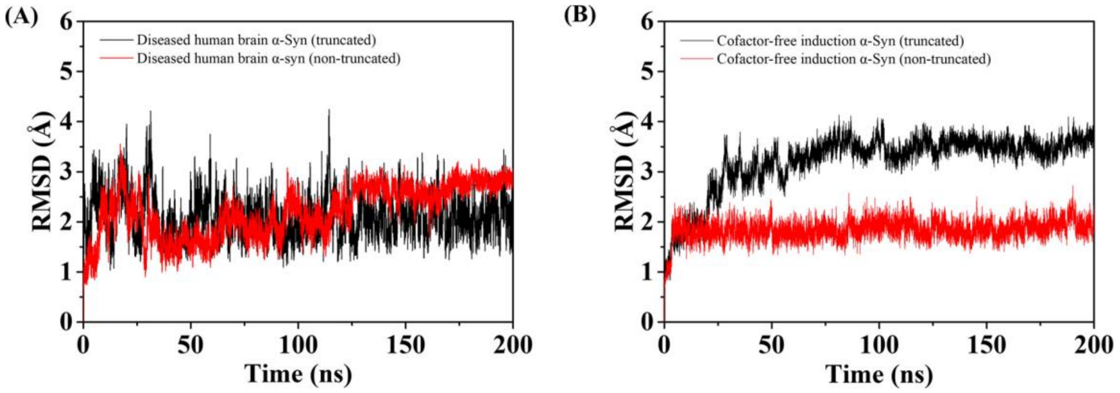

Figure S2. Convergence verification. (A) The backbone RMSDs of diseased human brain α-Syn fibrils as a function of time in truncated structure (black) and non-truncated structure (red). (B) The backbone RMSDs of cofactor-free induction α-Syn fibrils as a function of time in truncated structure (black) and non-truncated structure (red).

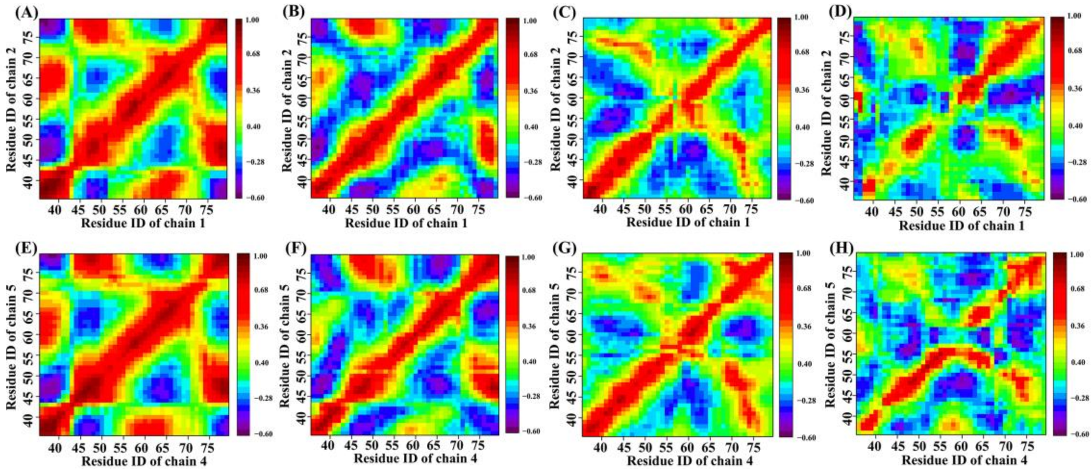

Figure S3. DCCM analysis from converged trajectories of the CMD simulation. (A,B) Chain-1/2 of non-truncated and truncated structures in human brain systems. (C,D) Chain-1/2 of non-truncated and truncated structures in cofactor-free system. (E,F) Chain-4/5 of non-truncated and truncated structures in human brain system. (G,H) Chain-4/5 of non-truncated and truncated structures in cofactor-free system. Red represents a strong interaction between residues, and blue depicts a weak interaction between residues.

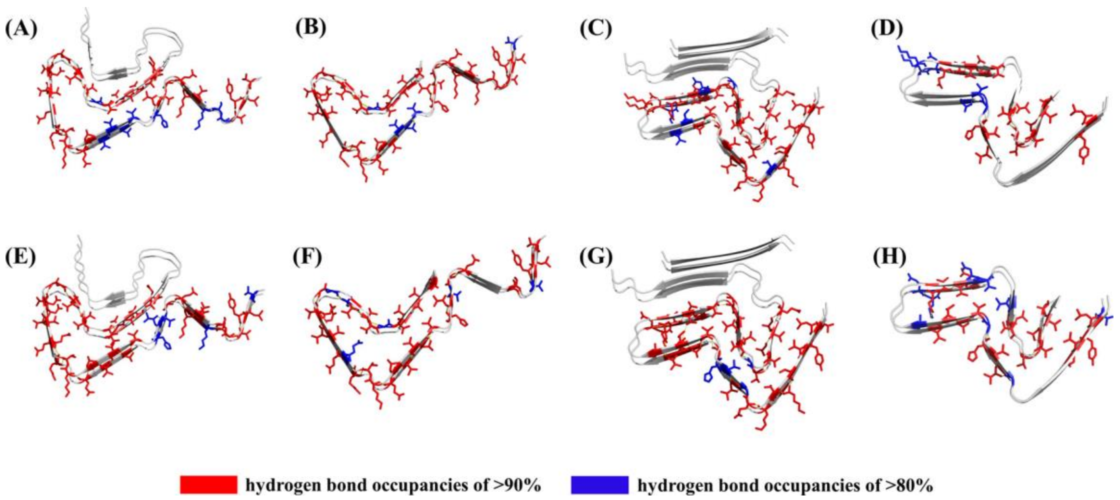

Figure S4. Backbone hydrogen bonds analysis from CMD simulation. (A,B) Chain-1/2 of non-truncated and truncated structures in human brain system. (C,D) Chain-1/2 of non-truncated and truncated structures in cofactor-free system. (E,F) Chain-4/5 of non-truncated and truncated structures in human brain system. (G,H) Chain-4/5 of non-truncated and truncated structures in the cofactor-free system. Red and blue represent the hydrogen bond occupancies of >90 and >80%, respectively.

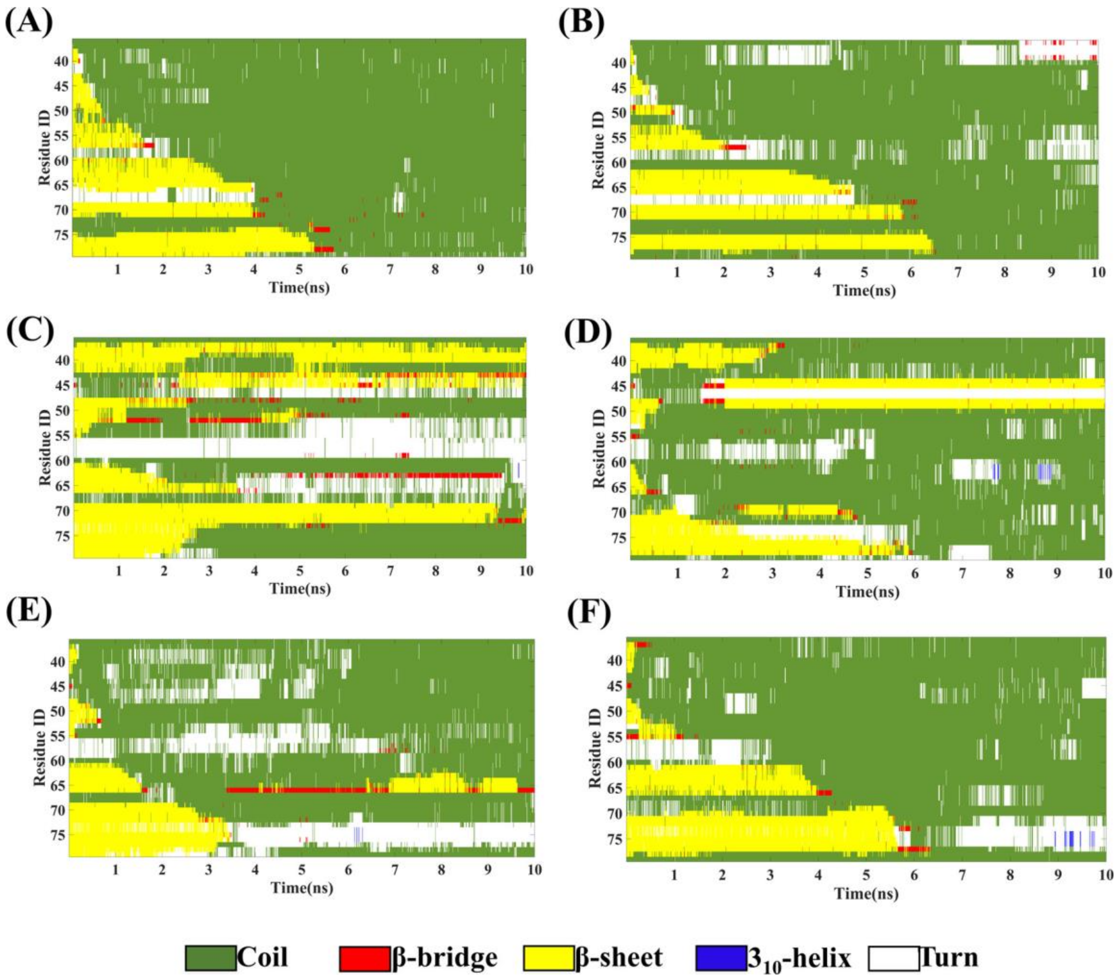

Figure S5. Changes in the secondary structure as a function of time. (A,B) Chain-1 of non-truncated structure and truncated in human brain system. (C,D) Chain-1 of non-truncated structure and truncated structures in cofactor-free system in path1. (E,F) Chain-1 of non-truncated structure and truncated structures in cofactor-free system in path2.

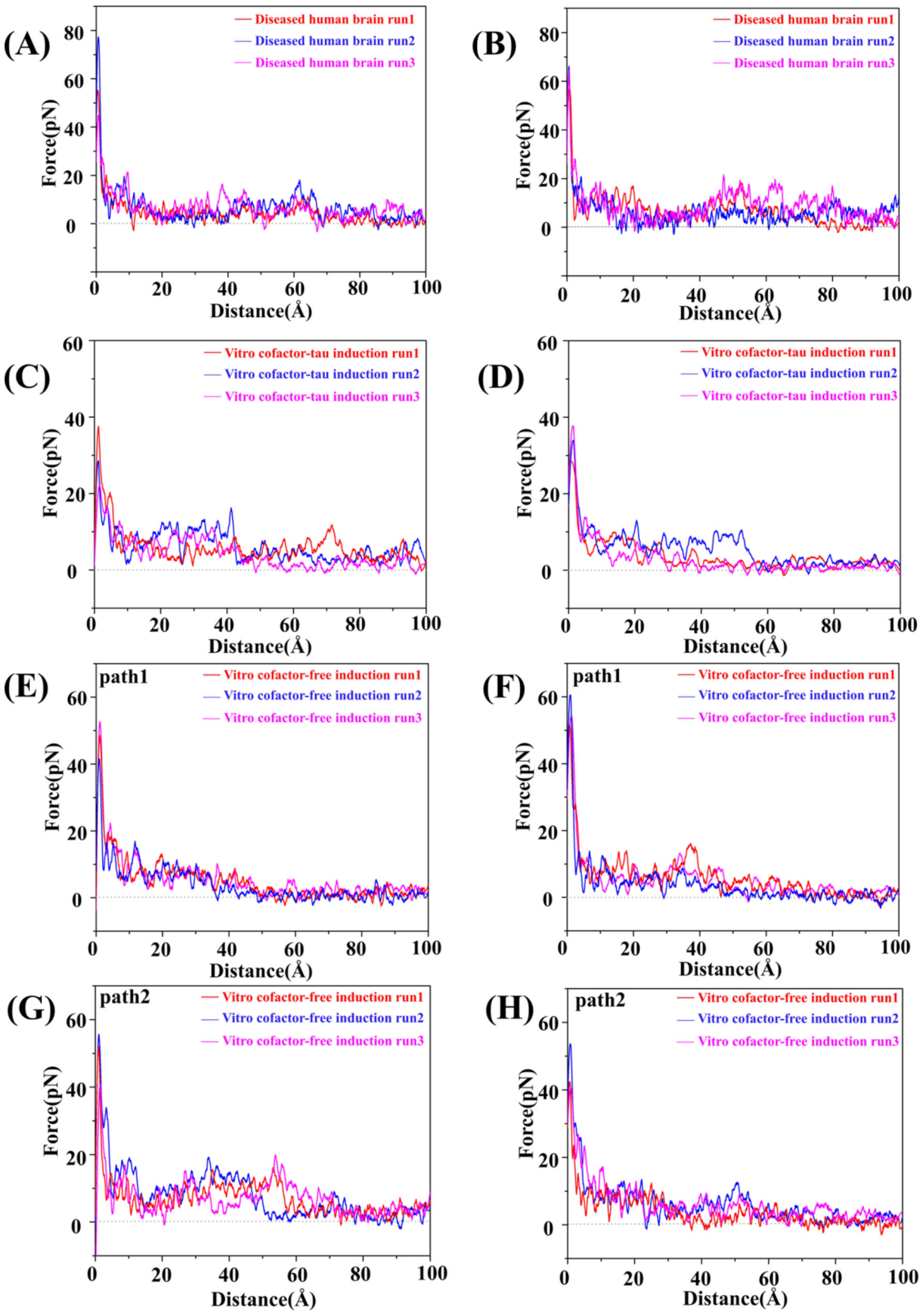

Figure S6. The changes of force over the reaction coordinate. (A,B) Chain-1 and Chain-5 of α-Syn fibrils in diseased human brains. (C,D) Chain-1 and Chain-4 of α-Syn fibrils in the cofactor-tau induction. (E,F) Chain-1 and Chain-5 of α-Syn fibrils in the cofactor-free induction in the path1. (G,H) Chain-1 and Chain-5 of α-Syn fibrils in the cofactor-free induction in the path2.

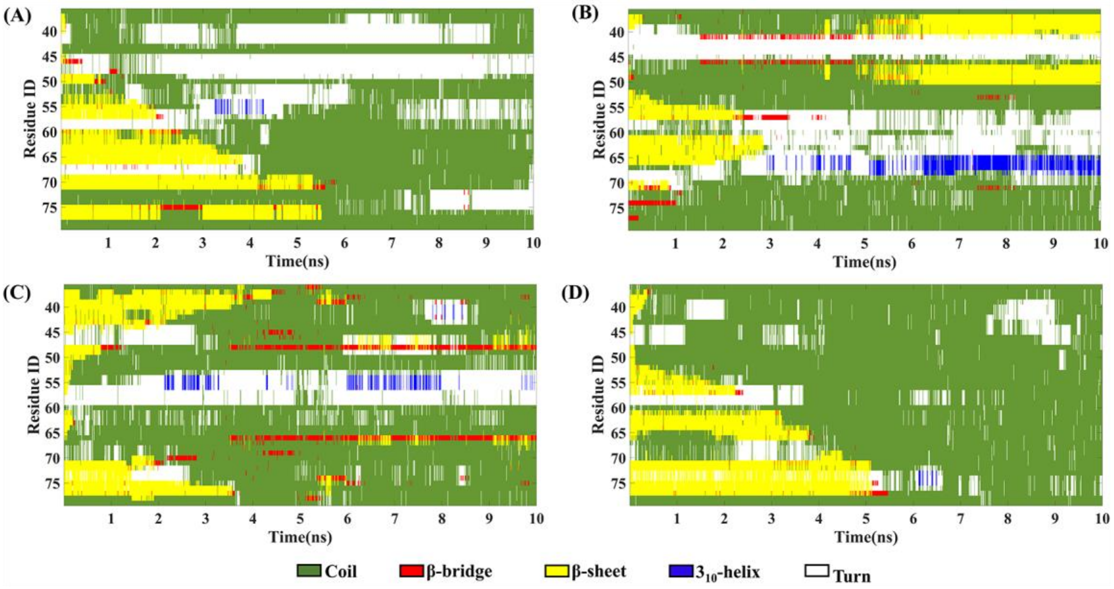

Figure S7. Changes in the secondary structure as a function of time. (A) Chain-5 of α-Syn fibrils in diseased human brain systems. (B) Chain-4 of α-Syn fibrils in the cofactor-tau induction. (C) Chain-5 of α-Syn fibrils in the cofactor-free induction in the path1. (D) Chain-5 of α-Syn fibrils in the cofactor-free induction in the path2.

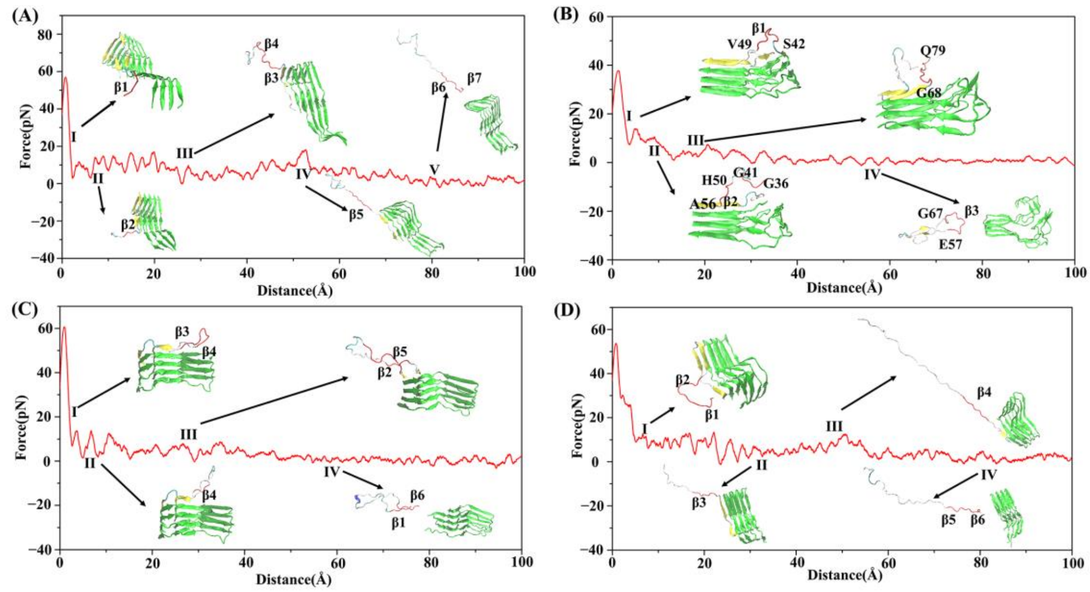

Figure S8. Changes of the force over the reaction coordinate. (A) Chain-5 of α-Syn fibrils in diseased human brain systems. (B) Chain-4 of α-Syn fibrils in the cofactor-tau induction. (C) Chain-5 of α-Syn fibrils in the cofactor-free induction in the path1. (D) Chain-5 of α-Syn fibrils in the cofactor-free induction in the path2. I−IV represents the five stages of dissociation. The arrow points to the representative conformation of each stage.

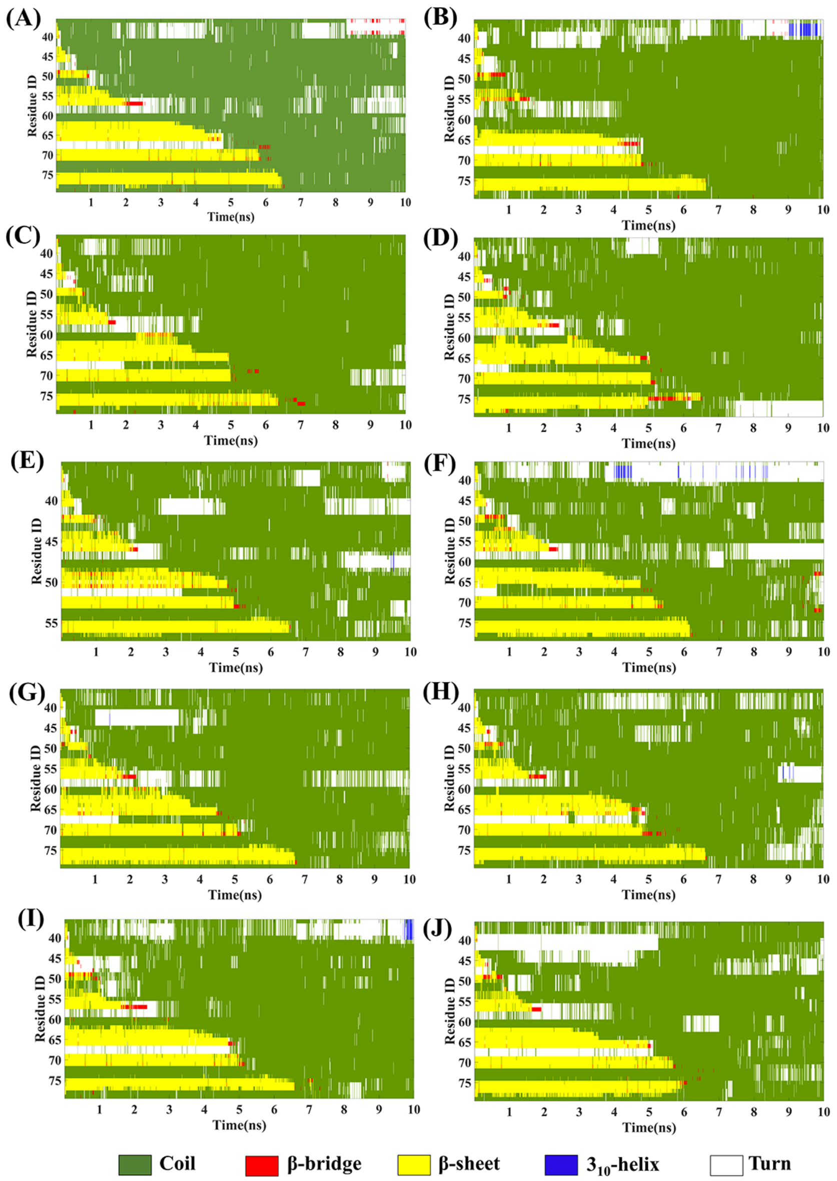

Figure S9. Changes in the secondary structure as a function of time. (A–J) represent run1-run10 on Chain 1 of α-Syn fibrils in diseased human brains.

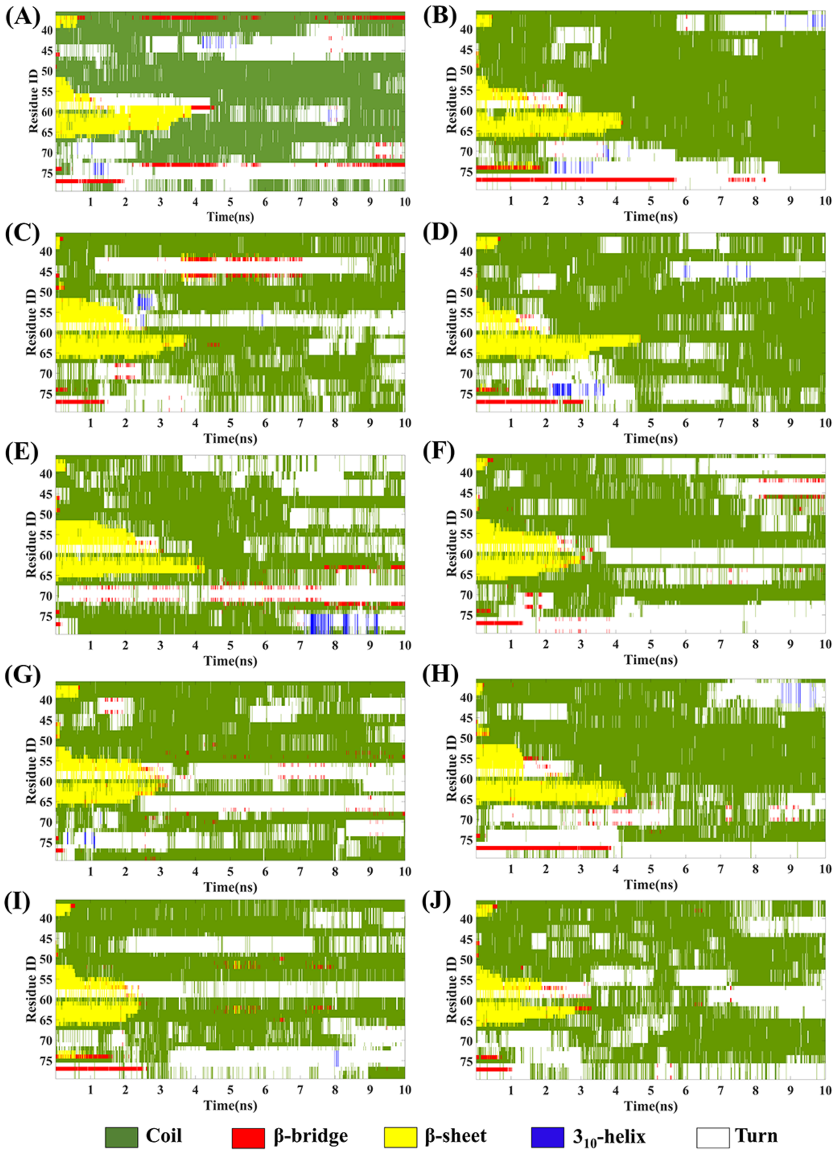

Figure S10. Changes in the secondary structure as a function of time. (A–J) represent run1-run10 on Chain 1 of α-Syn fibrils in the cofactor-tau induction model.

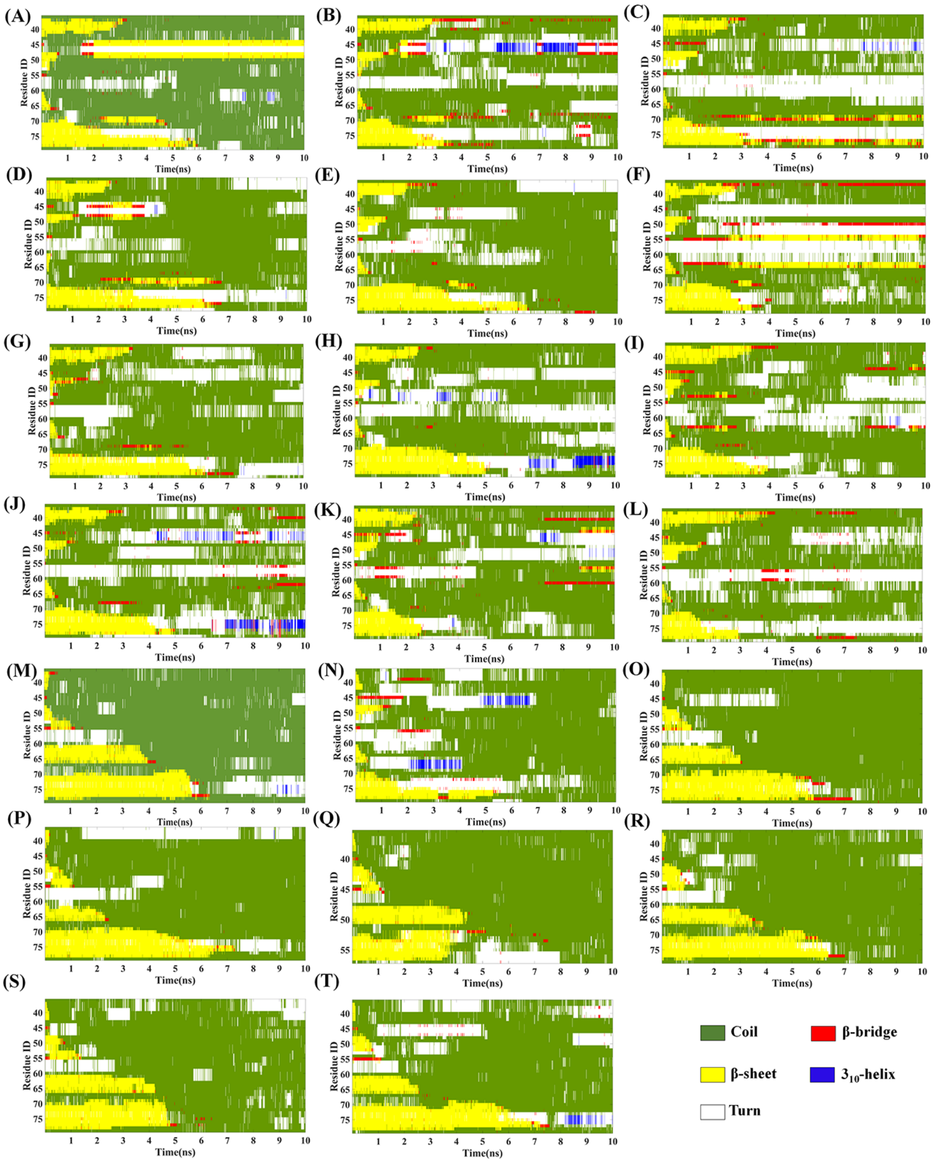

Figure S11. Changes in the secondary structure as a function of time. (A–L) represent run1-run12 on Chain 1 of α-Syn fibrils in the cofactor-free induction in the path 1. (M–T) represent run1-run8 on Chain 1 of α-Syn fibrils in the cofactor-free induction in the path 2.

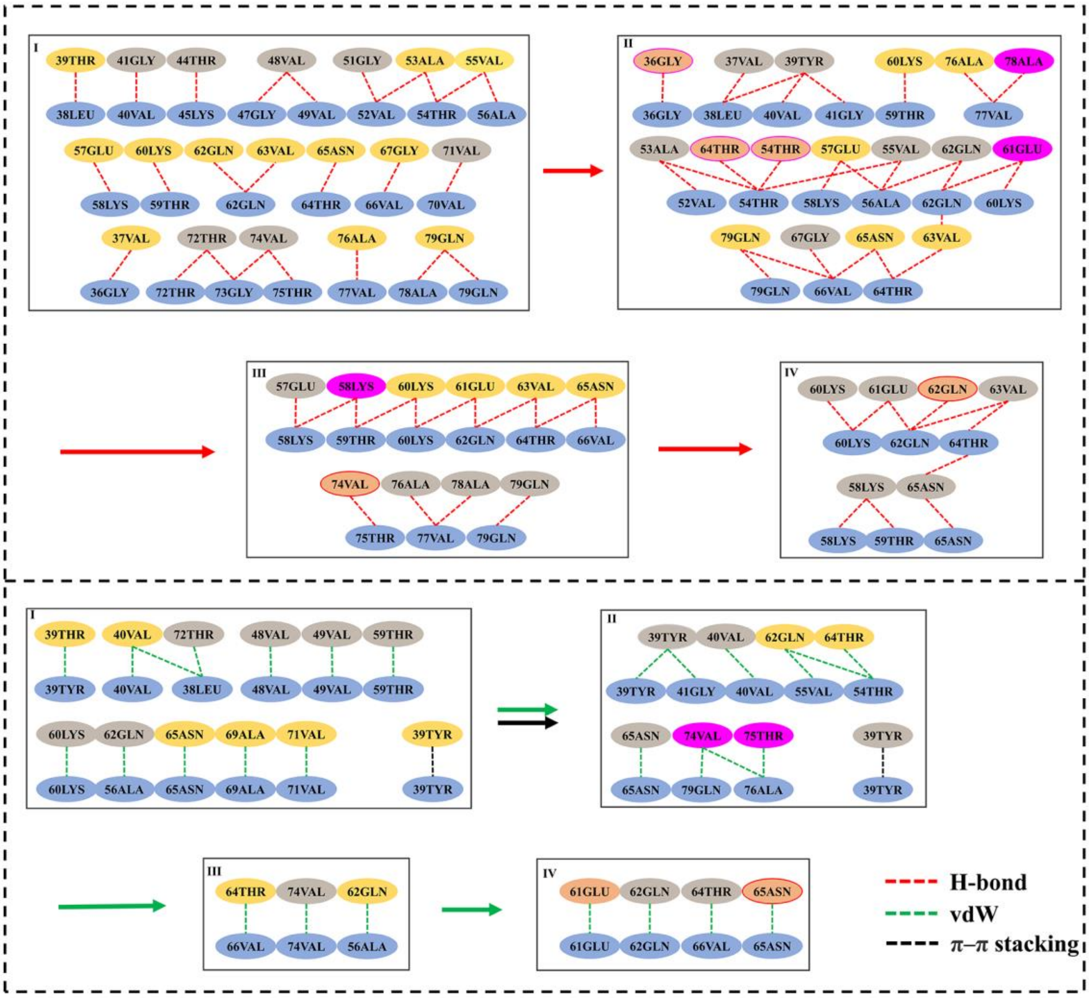

Figure S12. The residue interaction network analysis for cofactor-tau system.

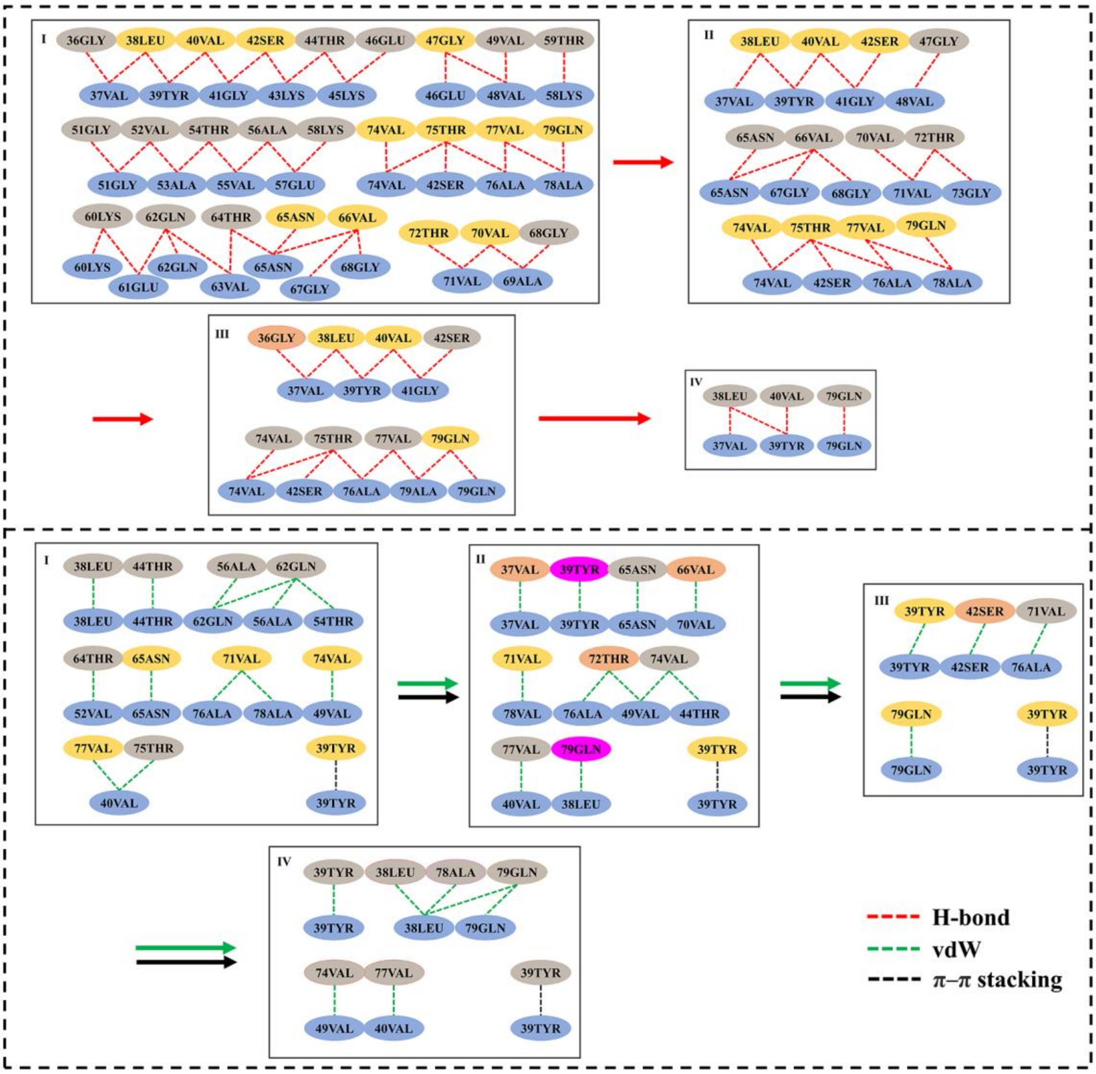

Figure S13. The residue interaction network analysis for cofactor-free system in path1.

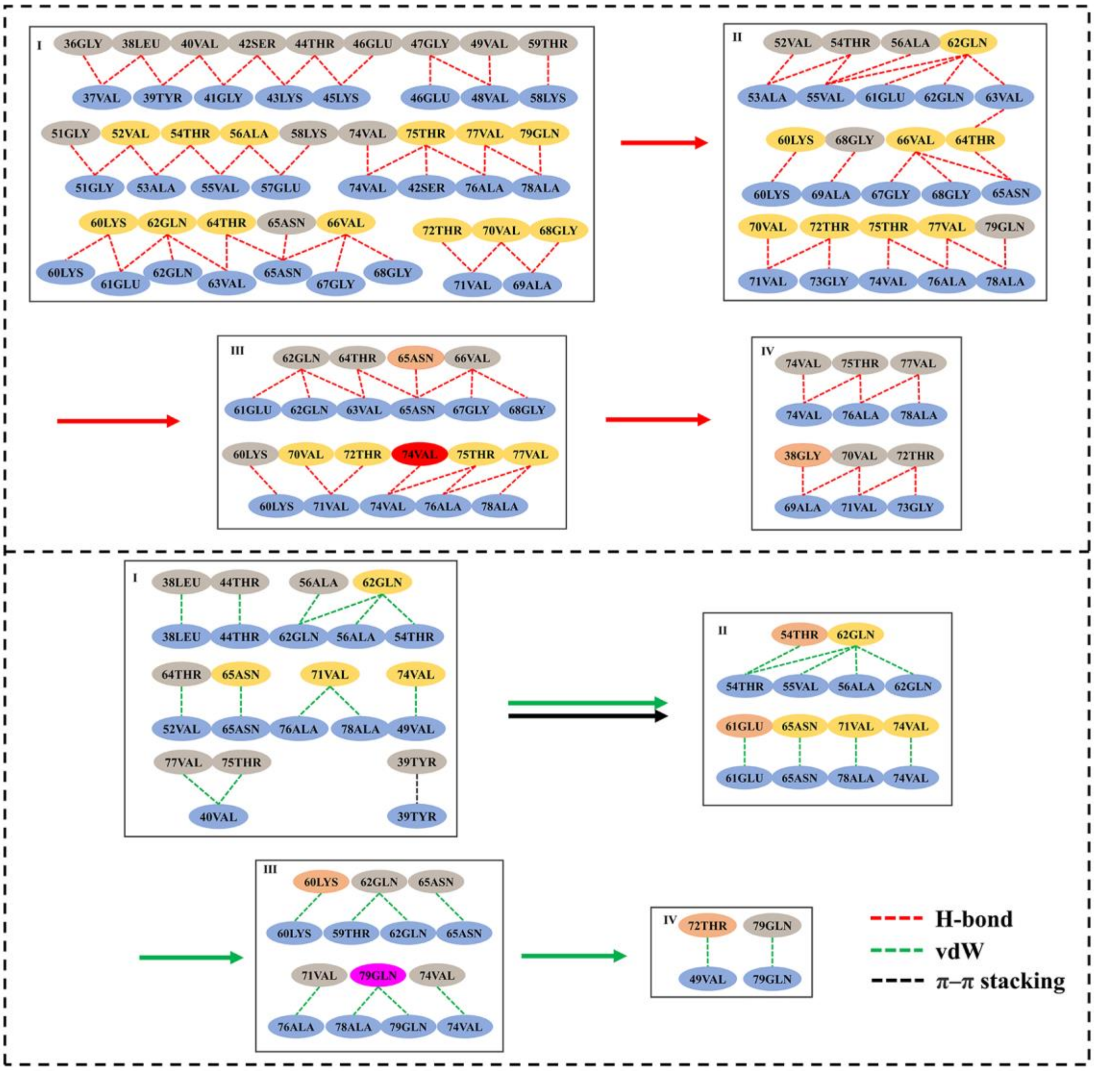

Figure S14. The residue interaction network analysis for cofactor-free system in path2.
